# The Increased Endogenous Sulfur Dioxide Acts as a Compensatory Mechanism for the Downregulated Endogenous Hydrogen Sulfide Pathway in the Endothelial Cell Inflammation

**DOI:** 10.3389/fimmu.2018.00882

**Published:** 2018-04-30

**Authors:** Da Zhang, Xiuli Wang, Xiaoyu Tian, Lulu Zhang, Guosheng Yang, Yinghong Tao, Chen Liang, Kun Li, Xiaoqi Yu, Xinjing Tang, Chaoshu Tang, Jing Zhou, Wei Kong, Junbao Du, Yaqian Huang, Hongfang Jin

**Affiliations:** ^1^Department of Pediatrics, Peking University First Hospital, Beijing, China; ^2^Animal Center, Peking University First Hospital, Beijing, China; ^3^Key Laboratory of Green Chemistry and Technology, Ministry of Education, College of Chemistry, Sichuan University, Chengdu, China; ^4^State Key Laboratory of Natural and Biomimetic Drugs, Beijing Key Laboratory of Molecular Pharmaceutics and New Drug Delivery Systems, School of Pharmaceutical Sciences, Peking University, Beijing, China; ^5^Department of Physiology and Pathophysiology, Peking University Health Science Centre, Beijing, China; ^6^Key Laboratory of Molecular Cardiology, Ministry of Education, Beijing, China

**Keywords:** endothelial cells, inflammation, sulfhydration, H_2_S, SO_2_

## Abstract

Endogenous hydrogen sulfide (H_2_S) and sulfur dioxide (SO_2_) are regarded as important regulators to control endothelial cell function and protect endothelial cell against various injuries. In our present study, we aimed to investigate the effect of endogenous H_2_S on the SO_2_ generation in the endothelial cells and explore its significance in the endothelial inflammation *in vitro* and *in vivo*. The human umbilical vein endothelial cell (HUVEC) line (EA.hy926), primary HUVECs, primary rat pulmonary artery endothelial cells (RPAECs), and purified aspartate aminotransferase (AAT) protein from pig heart were used for *in vitro* experiments. A rat model of monocrotaline (MCT)-induced pulmonary vascular inflammation was used for *in vivo* experiments. We found that endogenous H_2_S deficiency caused by cystathionine-γ-lyase (CSE) knockdown increased endogenous SO_2_ level in endothelial cells and enhanced the enzymatic activity of AAT, a major SO_2_ synthesis enzyme, without affecting the expressions of AAT1 and AAT2. While H_2_S donor could reverse the CSE knockdown-induced increase in the endogenous SO_2_ level and AAT activity. Moreover, H_2_S donor directly inhibited the activity of purified AAT protein, which was reversed by a thiol reductant DTT. Mechanistically, H_2_S donor sulfhydrated the purified AAT1/2 protein and rescued the decrease in the sulfhydration of AAT1/2 protein in the CSE knockdown endothelial cells. Furthermore, an AAT inhibitor l-aspartate-β-hydroxamate (HDX), which blocked the upregulation of endogenous SO_2_/AAT generation induced by CSE knockdown, aggravated CSE knockdown-activated nuclear factor-κB pathway in the endothelial cells and its downstream inflammatory factors including ICAM-1, TNF-α, and IL-6. In *in vivo* experiment, H_2_S donor restored the deficiency of endogenous H_2_S production induced by MCT, and reversed the upregulation of endogenous SO_2_/AAT pathway *via* sulfhydrating AAT1 and AAT2. In accordance with the results of the *in vitro* experiment, HDX exacerbated the pulmonary vascular inflammation induced by the broken endogenous H_2_S production in MCT-treated rat. In conclusion, for the first time, the present study showed that H_2_S inhibited endogenous SO_2_ generation by inactivating AAT *via* the sulfhydration of AAT1/2; and the increased endogenous SO_2_ generation might play a compensatory role when H_2_S/CSE pathway was downregulated, thereby exerting protective effects in endothelial inflammatory responses *in vitro* and *in vivo*.

## Introduction

Hydrogen sulfide (H_2_S), a new member of gaseous signal molecule family, has been found as a metabolic end product of sulfur-containing amino acids and to be involved in various physiologic and pathophysiologic processes since the end of the last century (1, 2). Cystathionine-γ-lysase (CSE) is regarded as a predominant H_2_S-generating enzyme in the cardiovascular tissues, and H_2_S is generated with the substrates of cystathionine or cysteine, catalyzed by CSE (3, 4). The regulatory effect of endogenous H_2_S on the endothelial cell function attracted great attention because of the importance of endothelial cells in the vascular injury and repair. H_2_S was reported to stimulate the proliferation and migration of endothelial cells, promote endothelial cell angiogenesis, inhibit the endothelial cell inflammation, protect mitochondrial function, and mediate endothelial-dependent vasorelaxation, etc (5–8). Plenty of research demonstrated that H_2_S protected the endothelial cells against various insults from hypoxia, high-salt, high-glucose, angiotensin II, and tumor necrosis factor-α (TNF-α), and so on (7, 9–12). Impaired endogenous H_2_S production, bioavailability, and its function were involved in the pathogenesis of endothelium dysfunction-related diseases including hypertension, vascular complication of diabetes, atherosclerosis, restenosis, and aging, etc (13–15).

Recently, sulfur dioxide (SO_2_), a brother of H_2_S, attracted more and more concerns in the field (16, 17). SO_2_ was found to be endogenously generated from the enzymatic reaction catalyzed by aspartate amino transferase (AAT) in the metabolic pathway of sulfur-containing amino acids (18). Endogenous SO_2_/AAT pathway was discovered to exist in the endothelium, vascular smooth muscles, fibroblasts, cardiac myocytes, adipocyte, and alveolar epithelial cells and play an important role in the cardiovascular homeostasis (19–24). Our research group firstly put forward the hypothesis that endogenous SO_2_ might be the fourth gaseous signal molecule involved in the regulation of cardiovascular system (25). Endogenous SO_2_ was discovered to promote the nitric oxide production and enhance the nitric oxide-induced vasodialation (26). It could protect against acute lung injury induced by limb ischemic/reperfusion (I/R) or by lipopolysaccharide or by oleic acid in rats (27–29). Moreover, AAT1 overexpression could alleviate the lung inflammatory response caused by oleic acid in a mice model of acute lung injury (29).

Collectively, both H_2_S and SO_2_ are generated from the same metabolic pathway in the similar origin tissues and exert similar biological effect (24, 29–33). For instance, Xiao et al. discovered that H_2_S mitigated cardiomyocyte injury caused by hypoxic-reoxygenation *via* decreasing autophagy (30), while Chen et al. demonstrated that SO_2_ also alleviated myocardial hypertrophy by inhibiting Ang II-activated autophagy in mice (31). Furthermore, the two gasotransmitters sometimes share the same signaling pathway, and even the same target residue. The activation of PI3K/Akt pathway mediated the protective effect of H_2_S preconditioning on the cerebral I/R injury (32). Meanwhile, it was involved in SO_2_ preconditioning-induced protection against myocardial I/R injury (24). H_2_S can inactivate inflammatory response by inhibiting the phosphorylation and nuclear translocation of NF-κB p65 *via* sulfhydrating NF-κB p65 cysteine 38 (33), whereas SO_2_ suppresses inflammatory response by sulfenylating NF-κB p65 at the same residue (29).

So, here comes the question that what is the significance of the coexistence of H_2_S and SO_2_ in the biologic tissues. Li and Luo et al. found that SO_2_ increased endogenous H_2_S production in the development of artherosclerosis and pulmonary hypertension, and the upregulation of endogenous H_2_S pathway might be one of protective mechanisms responsible for endogenous SO_2_ (23, 34). However, the impact of endogenous H_2_S on the endogenous SO_2_ production and its significance have been unclear. In the present study, we attempted to construct an endogenous H_2_S-defiency endothelial cell inflammation model by transfecting lentivirus-containing CSE shRNA using human umbilical vein endothelial cell (HUVEC) line (EA.hy926), investigate the effect of endogenous H_2_S on the endothelium-derived SO_2_ generation and explore its significance in the development of inflammatory response induced by the H_2_S/CSE deficiency. In addition, we also used the primary HUVECs, rat pulmonary artery endothelial cells (RPAECs) and rats with pulmonary vascular inflammation in the study to verify the effect of H_2_S on the endogenous SO_2_ production and its implication.

## Materials and Methods

### Cell Culture

The HUVEC line (EA.hy926) was purchased from China Infrastructure of Cell Line Resources Center, China. The cells were grown in Dulbecco’s modified Eagle’s medium (DMEM) supplemented with 10% fetal bovine serum (FBS), 1% streptomycin, and 1% penicillin (Gibco, USA). Primary HUVECs were kindly provided by professor Jing Zhou, Peking University Health Science Center, Beijing, China, and RPAECs (PriCells, Wuhan, China) were cultured in the specialized endothelial cell medium (PriCells, Wuhan, China) supplemented with 10% FBS and 100 IU/mL penicillin-streptomycin. The endothelial cells were maintained in a humidified atmosphere of 5% CO_2_ at 37°C.

CSE knockdown endothelial cells were obtained by infecting lentivirus containing CSE shRNA plus green fluorescent protein (GFP) cDNA (Cyagen, China). For the purpose of determining the appropriate concentration of lentivirus used for the treatment, the cells were seeded in 6-well plate, grown to 60–70% confluence, and transfected with different viral titers of lentivirus (1 × 10^4^ to 2 × 10^5^ TU/mL). After 12 h of the infection, freshly completed culture medium was replaced. After another 72 h, the green fluorescence of GFP was observed in the successfully transfected cells under fluorescence microscope. Moreover, the protein expression of CSE in the cells was detected by western blot and the H_2_S level in cell supernatant was detected by H_2_S-selective sensor. The screening results demonstrated that the appropriate concentration of lentivirus containing CSE shRNA plus GFP cDNA was 1 × 10^5^ TU/mL (Figure S1 in Supplementary Material). The endothelial cells were seeded in T25 flasks and infected with lentiviral CSE shRNA (1 × 10^5^ TU/mL) at 60–70% confluency. G418 antibiotics (200 µg/mL) was used for EA.hy926 cell screening for 2 week and G418 antibiotics (300 µg/mL) was used for primary HUVECs and primary RPAECs screening for 1 week. At the same time, vehicle lentivirus was used to infect the endothelial cells as the control according to the same protocol.

To explore the effect of endogenous H_2_S deficiency on the SO_2_/AAT pathway in the endothelial cell and its mechanism, cells were randomly divided into vehicle group, CSE shRNA group, and CSE shRNA plus H_2_S group. Cells in the CSE shRNA + H_2_S group were pretreated with 200 µM of H_2_S donor sodium hydrosulfide hydrate (NaHS) for 24 h. Cells in the vehicle group and CSE shRNA group were incubated with equal volume of ddH_2_O. NaHS was freshly dissolved in ddH_2_O.

To investigate the significance of the increased endogenous SO_2_ generation in the endothelial cell inflammation caused by CSE knockdown, cells were divided into vehicle group, CSE shRNA group, and CSE shRNA + L-aspartate-β-hydroxamate (HDX) group. Cells in the CSE shRNA + HDX group were pretreated with 200 µM HDX for 24 h. Cells in the control group and CSE shRNA infected group were incubated with equal volume of ddH_2_O. HDX is an inhibitor of AAT and freshly prepared.

### Animal Preparation and Grouping

All animal care and experimental procedures complied strictly with the Animal Management Rule of the Ministry of Health of the People’s Republic of China (Documentation 55, 2001). The protocol was specifically approved by the Animal Research Ethics Committee of Peking University First Hospital (permit number 201215 and 201326).

Eighteen male Wistar rats provided by the Animal Research Committee of the First Hospital, Peking University, weighing 160 ± 20 g, were randomly divided into three groups (*n* = 6 each group): control group, monocrotaline (MCT) group, and MCT + H_2_S group. On the first day, the rats of MCT and MCT + H_2_S groups were administered with MCT (60 mg/kg) by intraperitoneal injection, while the rats of control group were injected with the same dose of saline (5, 35). The rats of MCT + H_2_S group were injected daily with the H_2_S donor, NaHS (56 µmol/kg), for 21 days, while the rats of the control and MCT groups were given the same dose of saline.

Another 21 male Wistar rats were divided into three groups (*n* = 7 each group): control group, MCT group, and MCT + HDX group. The rats in the MCT and MCT + HDX groups were administered with MCT (60 mg/kg) by intraperitoneal injection on day 1. The rats in the MCT + HDX group were given HDX orally at 25 mg/kg on days 0, 7, and 14 (36). The rats in the control group received the same dose of saline.

### Rat Pulmonary Artery Pressure Measured by Right Heart Catheterization

Rats were anesthetized *via* intraperitoneal injection of 0.5% sodium pentobarbital (0.1 mL/100 g) after 21 days of MCT challenge. The pulmonary artery pressure was measured *via* right heart catheterization as previously described (22). Briefly, the right external jugular vein was exposed and a catheter was guided through the superior vena cava, right atrium, and right ventricle into the pulmonary artery. The extracorporal end of the catheter was connected to a pressure sensor (BL-410, Chengdu TME Technology, China) to record the continuous changes of pulmonary artery pressure, including systolic pulmonary artery pressure, diastolic pulmonary artery pressure, and mean pulmonary artery pressure.

### Morphological Change of Pulmonary Arteries

The rat lung tissue was immersed in the 10% (wt/vol) paraformaldehyde for fixation and then embedded in paraffin. The lung tissue was sectioned at a thickness of 4 µm. The elastic fiber in the pulmonary artery was stained using the modified Weigert’s elastic fiber staining kit according to the manufacturer’s protocol (Leagene, Beijing, China). The internal and external elastic lamina were shown as dark-purple color under microscope.

### Western Blotting

The specific protein expression in the endothelial cell and lung tissues was detected by western blotting. After treatment, the cells and rat lung tissues were lysed in lysis buffer (50 mM Tris base, 150 mM NaCl, 1 mM EDTA, 0.25% sodium deoxycholate, 1% NP-40, protease inhibitor cocktail, PH 7.4) (37). Protein concentration was determined using Bradford kit. Equal amounts of proteins were boiled and separated using 8–15% SDS-PAGE, and transferred using electrophoresis to a nitrocellulose membrane (Amersham, USA). The primary antibody dilutions were 1:200 for CSE (Sigma, USA), 1:1,000 for AAT1 and AAT2 (Sigma, USA), 1:200 for ICAM-1 (Boster, China), 1:1,000 for NF-κB p65 and IκBα (CST, USA), 1:500 for p-NF-κB p65 and p-IκBα (CST, USA), 1:2,000 for β-actin (Santa Cruz, CA, USA), and 1:2,000 for GAPDH (Kangcheng, China). Horseradish peroxidase-conjugated secondary antibodies were used at a dilution of 1:3,000–1:5,000 (Sigma, USA). The bands were visualized using a chemiluminescence detection kit on the FluorChem M MultiFluor System (Proteinsimple, USA). The densitometric analysis of the bands was performed using AlphaEaseFC (Alpha, USA). All experiments were performed independently for at least three times.

### Measurement of H_2_S Level by an H_2_S-Selective Sensor

The H_2_S level in endothelial cell supernatant and rat lung tissues was measured using the free radical analyzer TBR4100 with an H_2_S-selective sensor (ISO-H_2_S-100, WPI, China) as previously described (38, 39). The rat lung homogenate was prepared by grinding with cold PBS buffer (pH 7.2, 0.01 M). Firstly, an H_2_S-selective sensor was polarized with PBS buffer (pH 7.2, 0.05 M) until a stable baseline current was reached, and then the calibration curve of pA–H_2_S concentration began to be plotted as follows. The sensor tip was immersed by 10 mm into 20 mL of PBS buffer solution-containing Na_2_S at different concentrations (0.5, 1, 4, 8, 16, and 32 µM) sequentially. Then the calibration curve was constructed by plotting the signal output (pA) against the concentration (μM) of H_2_S. Secondly, the sensor tip was immersed into each sample by 10 mm to detect the H_2_S content in the sample according the calibration curve of pA–H_2_S concentration. All experiments were performed independently for at least three times.

### Measurement of SO_2_ Level by High-Performance Liquid Chromatography (HPLC) Analysis

SO_2_ content in the supernatant of EA.hy926 cells, primary HUVECs and RPAECs supernatants, and rat lung tissues was examined by HPLC (Agilent 1100 series, Agilent Technologies, Palo Alto, CA, USA) as previously described (29). The rat lung homogenate was prepared by grinding with cold PBS buffer (pH 7.2, 0.01 M). In brief, the sample was mixed with 0.212 mM sodium borohydride in 0.05 M Tris–HCl (pH 8.5) and incubated at room temperature for 30 min; this mixture was subsequently combined with 70 mM monobromobimane in acetonitrile. Then, perchloric acid was added, followed by vortex mixing. After that, the mixtures were centrifuged at 12,400× *g* for 10 min, and the supernatant was neutralized by 2.0 M Tris and subsequently centrifuged again at 12,400× *g* for 10 min. Eventually, the neutralized supernatant was transferred and injected into an HPLC column. Sulfite-bimane adduct was detected by the excitation at 392 nm and the emission at 479 nm. All experiments were performed independently for at least three times.

### *In Situ* Detection of H_2_S by Fluorescent Probe

The H_2_S generation in the endothelial cells was *in situ* detected by H_2_S fluorescent probe kindly provided by professor Xinjing Tang, Peking University Health Science Center, Beijing, China, as described previously (5). The cells were cultured using Lab-Tek chambered coverglass (Thermo, USA), rinsed with PBS for twice before incubation with the H_2_S fluorescent probe, then subsequently incubated with H_2_S fluorescent probes (100 µM) for 30 min, and fixed with ice-cold 4% paraformaldehyde for 20 min. Immunofluorescent images were obtained using a confocal laser-scanning microscope (TCS SP5, Leica, Wetzlar, Germany). Green fluorescent indicates endogenous H_2_S in the cells and the fluorescent signal intensity was measured using Image J software (NIH, Bethesda, MD, USA). All experiments were performed independently for at least three times.

### *In Situ* Detection of Endogenous SO_2_ by Fluorescent Probe

The SO_2_ generation in endothelial cells was detected *in situ* by SO_2_ fluorescent probe kindly provided by Professor Kun Li, College of Chemistry of Sichuan University, Sichuan, China. The specificity and sensitivity of this probe were previously verified (40, 41). The cells were cultured using Lab-Tek chambered coverglass (Thermo, USA) and subsequently incubated with SO_2_ fluorescent probes (20 µM) for 30 min and then rinsed twice with PBS prior to fixation with ice-cold 4% paraformaldehyde for 20 min. Then, cells were rinsed twice with PBS, each for 5 min before testing. Immunofluorescent images were obtained using a confocal laser-scanning microscope (TCS SP5, Leica Microsystems, Wetzlar, Germany). Blue fluorescent indicates endogenous SO_2_ in the cells. The fluorescent signal intensity was measured using Image J software (NIH, Bethesda, MD, USA). All experiments were performed independently for at least three times.

### AAT Activity Detected by Colorimetry Assay

The activity of AAT in endothelial cells, purified AAT protein from pig heart, and rat lung tissues was tested by colorimetry assay (JianCheng, Nanjing, China) according to the manufacturer’s instructions as described previously (18). AAT catalyzes the transfer of amino group and keto group in α-ketoglutaric acid and aspartic acid to form glutamic acid and oxaloacetic acid. Oxaloacetic acid is then decarboxylated by itself to form pyruvic acid, and the latter was reacted with 2,4-dinitrophenylhydrazine to produce the 2,4-dinitrophenylhydrazone which shows a red–brown color in alkaline solution and can be detected by colorimetric method. Pyruvic acid solution (2 mM) was used as the standard to plot the standard curve. Endothelial cells were homogenized in PBS with an ice-water bath and centrifuged at 5,000× *g* for 10 min at 4°C to get the supernatant. Equivalent AAT purified proteins (0.375 µg, Sigma, USA) were incubated at different concentrations of H_2_S (100, 200, and 500 µM) or double distilled water for 2 hr in 37°C water bath. In the 200 µM H_2_S plus DTT treatment, purified AAT protein was pretreated with NaHS (200 µM) for 1 h and then incubated with 1 mM DTT for a further 1 h in the continuous presence of NaHS. The rat lung homogenate was prepared by grinding with cold PBS buffer (pH 7.2, 0.01 M). AAT activity was expressed as Carmen’s unit, which was calculated according to the standard curve after colorimetric determination. One unit of Carmen’s is defined as follows: NADH is oxidized to NAD^+^ by pyruvic acid generated from 1 mL of the sample within 1 min at 25°C in the total reaction capacity of 3 mL, which causes the absorbance deceased by 0.001 at 340 nm wavelength using a light path length of 1 cm. All experiments were performed independently for at least three times.

### S-Sulfhydrtation Detected by Biotin Switch Analysis

S-sulfhydration of AAT1 and AAT2 in EA.hy926 cell line, primary HUVECs, RPAECs, and rat lung tissues was detected by biotin switch assay as described previously (33, 42). Endothelial cells or rat lung tissues were homogenized in non-denaturing lysis buffer with protease inhibitors and centrifuged at 13,000× *g* for 20 min at 4°C. Supernatant reserved for sulfhydration analysis was incubated with blocking buffer (lysis buffer supplemented with 2.5% SDS and 20 mM S-methyl methanethiosulfonate) at 50°C for 30 min with continuous vortexing. The sample was added with acetone for removing S-methyl methanethiosulfonate at −20°C for 2 h. After acetone removal by centrifuge, the protein was resuspended in lysis buffer and incubated with EZ-link iodoacetyl-PEG2 biotin (10 mg/mL) at 4°C for 12 h. Biotinylated proteins were precipitated by UltraLink™ Immobilized Neutravidin™ for 4 h on a roller system (100 rpm) at 4°C and then washed three times with PBS. The sulfhydrated proteins were boiled with loading buffer without β-mercaptoethanol and centrifuged at 5,000× *g* for 10 min to get supernatant, and then subjected to western blot using 8% SDS-PAGE as described previously. All experiments were performed independently for at least three times.

### S-Sulfhydrtation Detected by Biotin Thiol Assay

S-sulfhydrtation of AAT1 and AAT2 in EA.hy926 cell line and purified AAT protein from pig heart was detected by the biotin thiol assay as described before (43). The schematic protocol is shown in Figure S2 in Supplementary Material. Cells were homogenized in non-denaturing lysis buffer with protease inhibitors and centrifuged at 13,000× *g* for 20 min at 4°C. The protein concentrations were determined by the BCA assay. Equal amount (1 mg) of total protein was incubated with 100 µM maleimide-PEG2-biotin (Thermo, USA) for 0.5 h on a roller system (100 rpm) at room temperature. Subsequently, the mixture was added with acetone at −20°C for 20 min. After washing for three times with 70% precooled acetone, the sample was centrifuged at 12,000× *g* for 5 min. The precipitate was resuspended in the buffer (0.1% SDS, 150 mM NaCl, 1 mM EDTA and 0.5% Triton X-100, 50 mM Tris–HCl, pH 7.5) mixed with streptavidin–agarose resin (Thermo, USA) and kept rotating overnight at 4°C. The beads were washed three times with PBS containing 0.5% Triton X-100 and centrifuged at 5,000× *g* for 5 min, and then the precipitate was mixed with 50 µL of loading buffer containing or not containing DTT (20 mM) with gentle shaking for 1.0 h on a roller system (100 rpm) at room temperature. After centrifugation at 5,000× *g* for 10 min, supernatant subjected to western blot using 8% SDS-PAGE as described previously. Equal amount (3 µg) of purified AAT protein (Sigma, USA) was incubated with NaHS (200 µM) for 2 h. The sulfhydrated AAT was separated and measured using the abovementioned protocol. All experiments were performed independently for at least three times.

### Expression of IκBα in Primary RPAECs Detected by Immunofluorescence

Immunofluorescent imaging was obtained using a confocal laser-scanning microscope (TCS SP5, Leica, Germany). Briefly, RPAECs were rinsed with PBS before the fixation with 4% paraformaldehyde. The RPAECs were then incubated with the anti-IκBα antibody (1:50, CST, USA) at 4°C overnight. RPAECs were subsequently incubated with the anti-mouse-FITC conjugated secondary antibody (Thermo, USA) at 37°C for 1 h. After washing, the slides were observed under confocal microscope (5). All experiments were performed independently for at least three times.

### Inflammatory Cytokine Levels Detected by Enzyme-Linked Immunosorbent Assay (ELISA)

Inflammatory cytokines including TNF-α, IL-6, and ICAM-1 in the cell supernatant and rat lung tissue homogenates were measured using ELISA kits (eBioscience, CA, USA). Recombinant TNF-α, IL-6, and ICAM-1 were used as standard substances. Samples and standard substances were incubated separately with an equal volume of diluent in an microplate coated with specific primary antibody at room temperature for 2 h using a shaker. Subsequently, the supernatant was removed, and the wells were rinsed with washing solution and dried. Horseradish peroxidase-conjugated primary antibody was then added to the wells and incubated for 1 h. After rinsing with washing solution, 100 µL of substrate solution was added to each well to develop the chromogenic reaction for 15 min. Then, 50 µL of stop solution was added to each well to stop the reaction. A standard curve was made by absorbance at 450 nm as the vertical axis and standard substance concentration as the horizontal axis. The concentrations of inflammatory cytokines in the samples were then calculated (5). The protein concentration of rat lung tissue homogenate was determined with Bradford kit and used for adjusting the content of cytokines in the rat lung tissue. All experiments were performed independently for at least three times.

### Statistical Analysis

Data are expressed as mean ± SEM. Comparisons among groups were analyzed by one-way ANOVA using SPSS 17.0 (SPSS Inc., USA). Means between groups with equal variance were analyzed by least-significance difference (LSD). When equal variance not assumed, means between groups were analyzed using Tamhane. *P* < 0.05 was considered statistically significant.

## Results

### Endogenous SO_2_ Production Was Increased in CSE Knockdown Endothelial Cells

**Figure 1:**
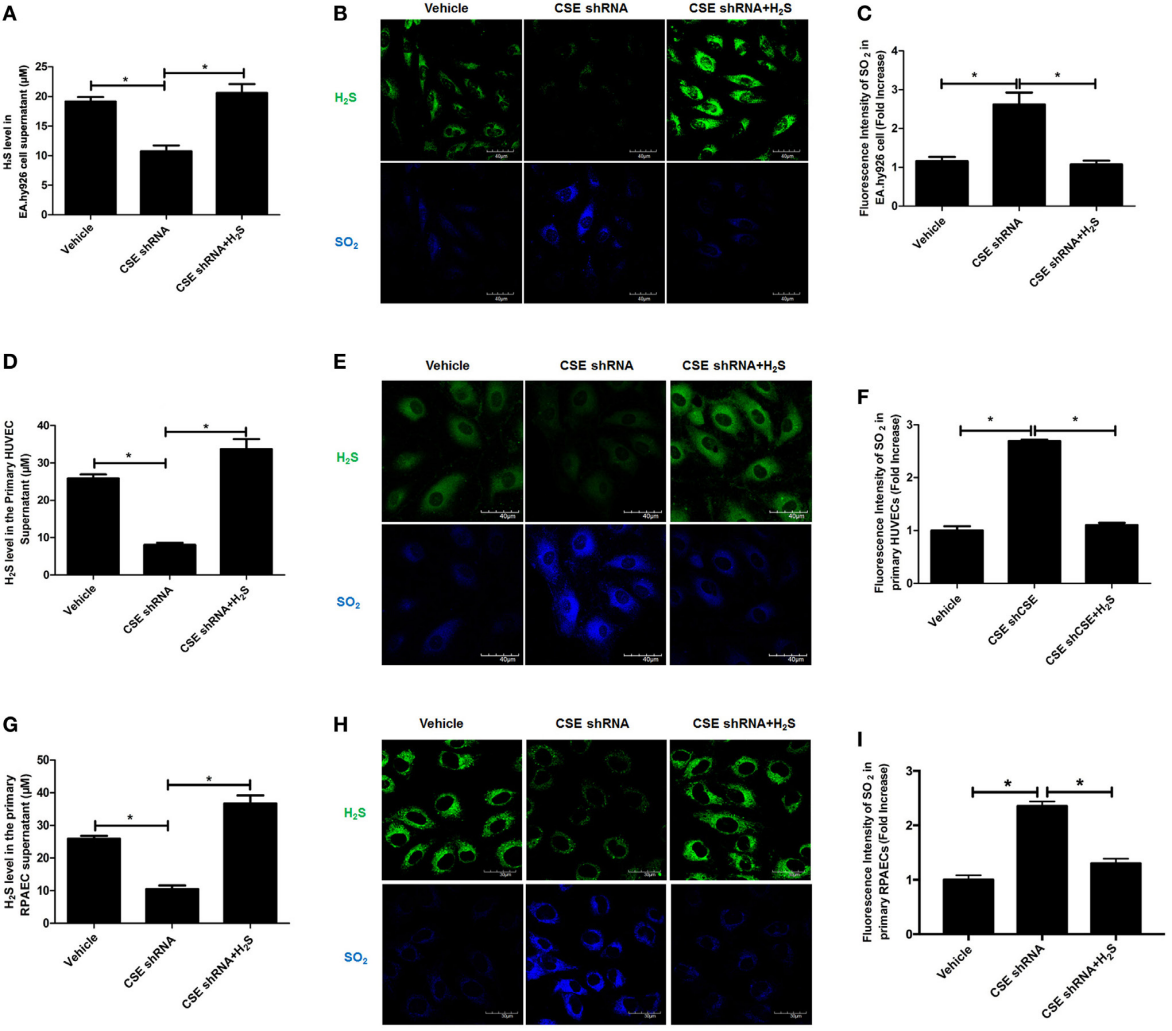
Endogenous SO_2_ production was increased in cystathionine-γ-lyase (CSE) knockdown endothelial cells. The EA.hy926 cells, primary human umbilical vein endothelial cells (HUVECs), and primary rat pulmonary artery endothelial cells (RPAECs) were transfected with vehicle lentivirus or lentivirus containing CSE shRNA, and then treated with or without H_2_S donor (200 µM). **(A)** The H_2_S level in cell supernatant was detected by H_2_S-selective sensor in EA.hy926 cell. **(B)** H_2_S generation in HUVECs was detected by *in situ* fluorescent H_2_S probe (green color), while SO_2_ generation in HUVECs was detected by *in situ* fluorescent SO_2_ probe (blue color) in EA.hy926 cell. **(C)** The blue fluorescence intensity indicating endogenous SO_2_ content was analyzed using Image J software in EA.hy926 cell. **(D)** The H_2_S level in cell supernatant was detected by H_2_S-selective sensor in primary HUVECs. **(E)** H_2_S generation in primary HUVECs was detected by *in situ* fluorescent H_2_S probe (green color), while SO_2_ generation in primary HUVECs was detected by *in situ* fluorescent SO_2_ probe (blue color). **(F)** The blue fluorescence intensity indicating endogenous SO_2_ content was analyzed using Image J software in primary HUVECs. **(G)** The H_2_S level in cell supernatant was detected by H_2_S-selective sensor in primary RPAECs. **(H)** H_2_S generation in primary RPAECs was detected by *in situ* fluorescent H_2_S probe (green color), while SO_2_ generation in primary RPAECs was detected by *in situ* fluorescent SO_2_ probe (blue color). **(I)** The blue fluorescence intensity indicating endogenous SO_2_ content was analyzed using Image J software in primary RPAECs. *P < 0.05. Data are expressed as means ± SEM, and all experiments were performed independently for at least three times.

For the purpose of revealing the effect of endogenous CSE/H_2_S pathway on endogenous SO_2_ production, EA.hy926 cell line was treated with CSE shRNA followed by H_2_S donor supplement. Compared with vehicle group, H_2_S level in cell supernatant was decreased in endothelial cells of CSE shRNA group, while H_2_S donor reimbursed the H_2_S deficiency caused by CSE knockdown (Figure 1A). In *in situ* fluorescent probe experiment, the data showed that CSE knockdown decreased the endogenous H_2_S level but promoted endogenous SO_2_ production (Figures 1B,C). Moreover, H_2_S donor NaHS inhibited the increase in endogenous SO_2_ level in the CSE knockdown EA.hy926 cells (Figures 1B,C). To further confirm the above result, we selected primary HUVECs and RPAECs in addition to EA.hy926 cells (Figures 1D–I). Interestingly, the results in both primary endothelial cells were accordant with that in the EA.hy926 cell. Compared with the vehicle group, SO_2_ level in both primary endothelial cells of CSE shRNA group was upregulated, while H_2_S donor blunted the effect of CSE knockdown on the endogenous SO_2_ level in both primary endothelial cells (Figures 1E,F,H,I). The results suggested that endogenous H_2_S suppressed the SO_2_ production in endothelial cells.

### The Protein Expression of AAT1 and AAT2 in Endothelial Cells Was Not Affected by CSE Knockdown

**Figure 2:**
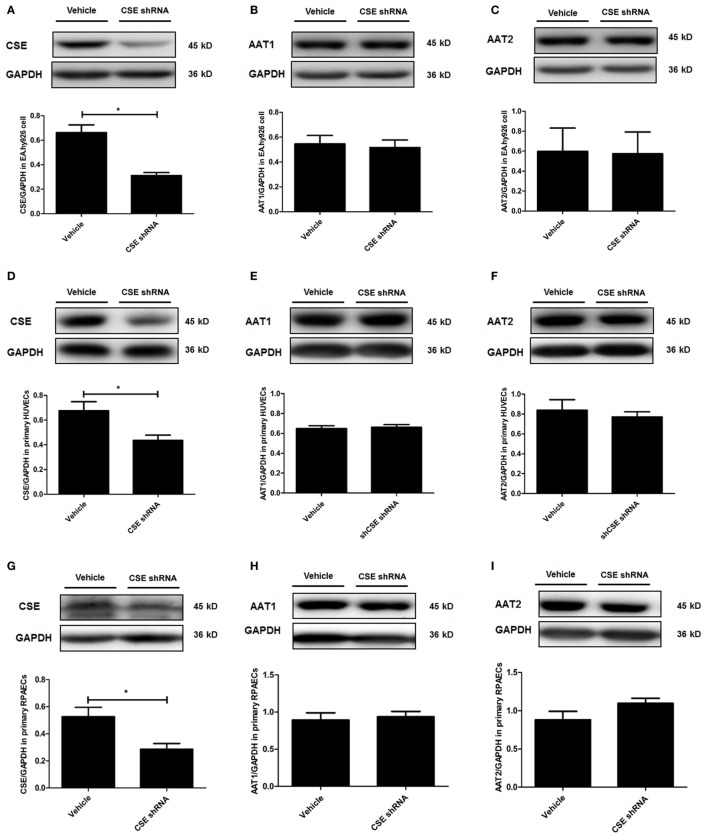
Cystathionine-γ-lyase (CSE) knockdown did not affect the expression of AAT1 and AAT2 in the endothelial cells. The EA.hy926 cells, primary human umbilical vein endothelial cells (HUVECs), and primary rat pulmonary artery endothelial cells (RPAECs) were transfected with vehicle lentivirus or lentivirus containing CSE shRNA. The expressions of CSE **(A)**, AAT1 **(B)**, and AAT2 **(C)** in the EA.hy926 cell were detected by western blot. The expressions of CSE **(D)**, AAT1 **(E)**, and AAT2 **(F)** in the primary HUVECs were detected by western blot. The expressions of CSE **(G)**, AAT1 **(H)**, and AAT2 **(I)** in the primary RPAECs were detected by western blot. *P < 0.05. Data are expressed as means ± SEM, and all experiments were performed independently for at least three times.

In order to elucidate the target on which endogenous CSE/H_2_S inhibited SO_2_ production, we first detected the protein expression of AAT1 and AAT2, the two key endogenous SO_2_ producing enzymes in EA.hy926 cells. Compared with vehicle group, the expression of CSE in the endothelial cells of CSE shRNA group was markedly decreased (Figure 2A). However, there was no difference in the expressions of AAT1 and AAT2 in the endothelial cells between vehicle group and CSE shRNA group (Figures 2B,C). Next, the same protocol of the experiments was done on both kinds of primary cells. Compared with the vehicle group, the expression of CSE was both downregulated in primary HUVECs and primary RPAECs (Figures 2D,G), while the expression of AAT1 and AAT2 was not affected by CSE knockdown (Figures 2E,F,H,I). The results proved that AAT1 and AAT2 protein expressions were not involved in the inhibitory effect of endogenous H_2_S/CSE on the endogenous SO_2_ production.

### The Endogenous H_2_S/CSE Inhibited the AAT Activity

**Figure 3:**
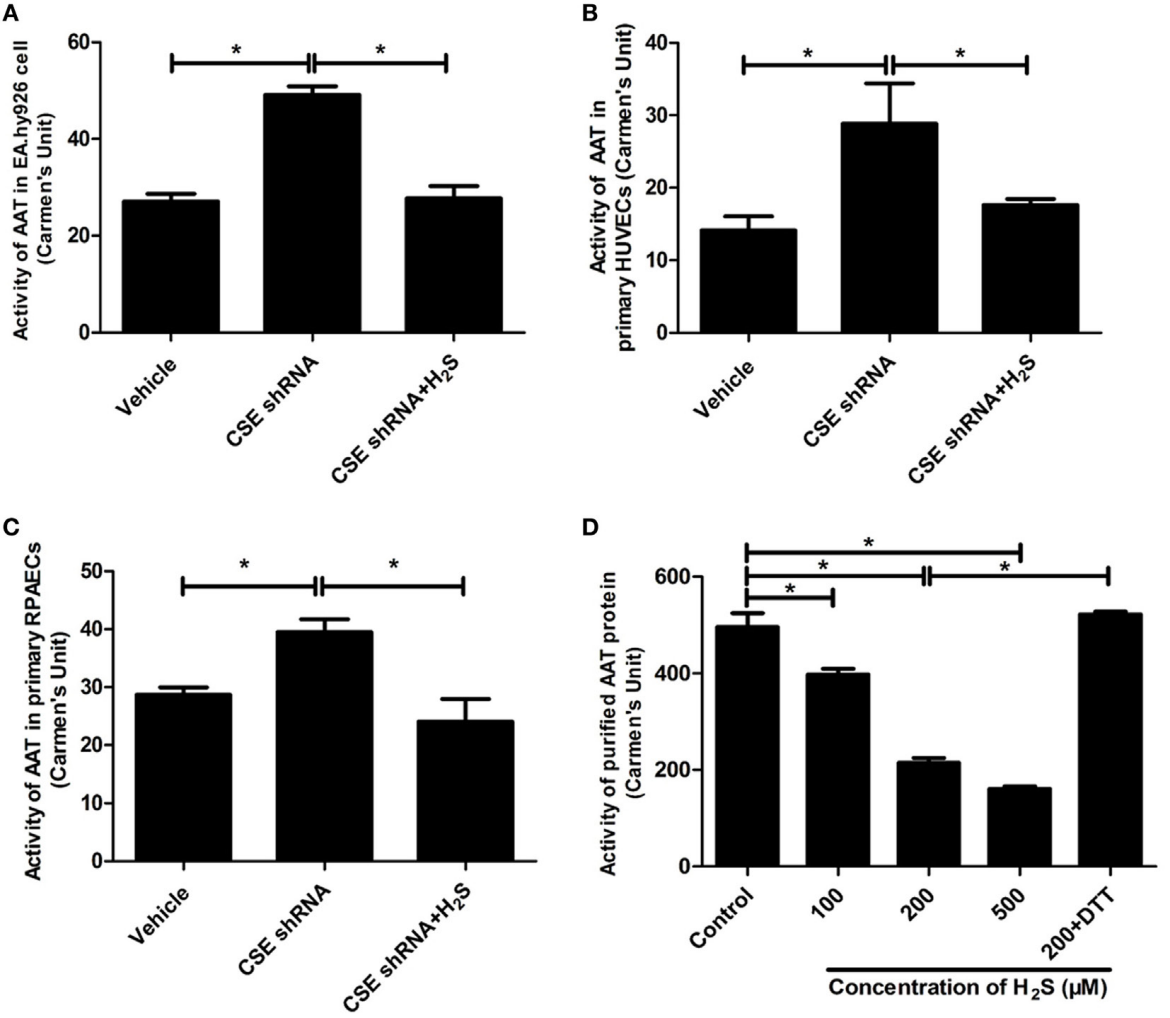
Endogenous H_2_S inhibited aspartate aminotransferase (AAT) activity in the endothelial cells and purified AAT protein. The activity of AAT in the EA.hy926 cells, primary human umbilical vein endothelial cells (HUVECs), primary rat pulmonary artery endothelial cells (RPAECs), and purified AAT protein were detected using colorimetric method. The endothelial cells were intervened by cystathionine-γ-lyase (CSE) knockdown, then pretreated with or without H_2_S donor (200 µM) for 24 h. **(A)** AAT activity in the EA.hy926 cells. **(B)** AAT activity in the primary HUVECs. **(C)** AAT activity in the primary RPAECs. **(D)** AAT activity of purified AAT protein. Different concentrations of H_2_S donor NaHS (100, 200, and 500 µM) were incubated with purified AAT protein from pig heart for 2 h. In the 200 µM H_2_S plus DTT treatment, purified AAT protein was pretreated with NaHS (200 µM) for 1 h, and then incubated with 1 mM DTT for a further 1 h in the continuous presence of NaHS. *P < 0.05. Data are expressed as means ± SEM, and all experiments were performed independently for at least three times.

Aspartate aminotransferase activity is another important element involved in the regulation of endogenous SO_2_ production. Therefore, we further detected the activity of AAT in the HUVECs and purified AAT protein. The result showed that activity of AAT was significantly increased in the CSE knockdown EA.hy926 cells. While compared with the vehicle cells, the exogenous supplementation of NaHS (200 µM) reversed the increase in the AAT activity caused by CSE knockdown (Figure 3A). The similar results were observed in both primary endothelial cells as shown in Figures 3B,C. Furthermore, NaHS (100–500 µM) directly inhibited the AAT activity in a concentration-dependent manner in purified AAT protein (Figure 3D), which further supported the direct inhibitory effect of H_2_S on the AAT activity.

### H_2_S S-Sulfhydrated AAT to Inhibit AAT Activity

**Figure 4:**
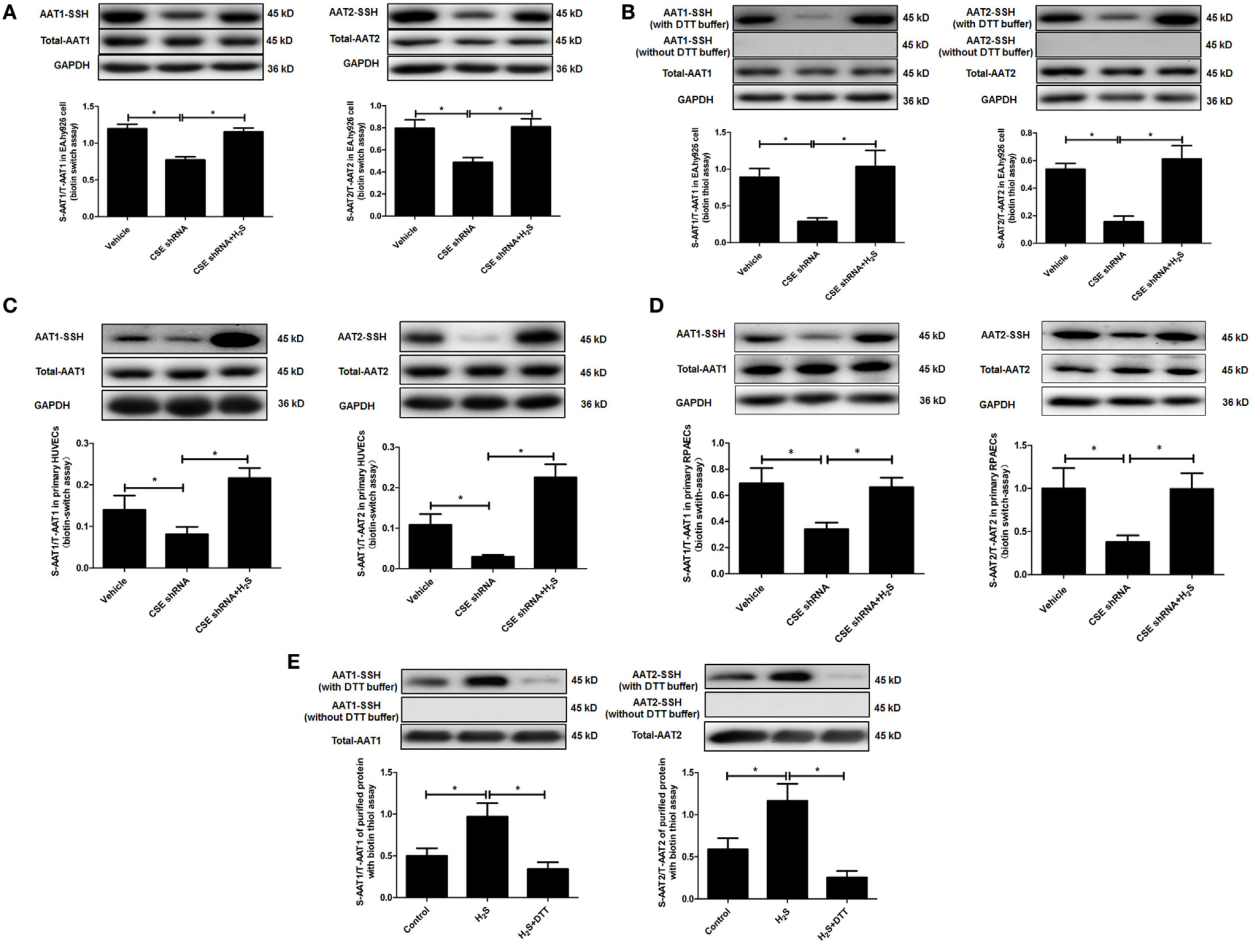
Endogenous H_2_S induced the sulfhydration of aspartate aminotransferase (AAT) in the endothelial cells and purified protein. **(A)** Sulfhydration of AAT1 and AAT2 in the EA.hy926 cell detected by biotin switch assay. **(B)** Sulfhydration of AAT1 and AAT2 in the in the EA.hy926 cell detected by biotin thiol assay. **(C)** Sulfhydration of AAT1 and AAT2 in the primary human umbilical vein endothelial cells (HUVECs) detected by biotin switch assay. **(D)** Sulfhydration of AAT1 and AAT2 in the primary rat pulmonary artery endothelial cells (RPAECs) detected by biotin switch assay. **(E)** Sulfhydration of purified AAT1 and AAT2 proteins from pig heart detected by biotin thiol assay. *P < 0.05. Data are expressed as means ± SEM, and all experiments were performed independently for at least three times.

In *in vitro* experiment, DTT, a thiol reductant, could reverse the impact of H_2_S on the AAT activity (Figure 3D), suggesting that the thiol group at the cysteine of AAT protein might be involved in the mechanisms by which H_2_S suppressed AAT activity. Considering that S-sulfhydration, a special posttranslation modification on the thiol group at the cysteine, was reported to participate in the wide biological effects of H_2_S, we detected the S-sulfhydration of AAT in the EA.hy926 cell, using modified biotin switch assay. The data showed that compared with vehicle group, S-sulfhydration of AAT1 and AAT2 was sharply reduced in EA.hy926 cell of CSE shRNA group, while the supplementation of NaHS significantly reversed the decrease in the S-sulfhydration of AAT in the EA.hy926 cell caused by CSE knockdown (Figure 4A). Furthermore, we also used another method for detecting S-sulfhydration, known as biotin thiol assay. The results in Figure 4B are in accordance with those shown in Figure 4A, suggesting that H_2_S can sulfhydrate AAT1 and AAT2.

Next, we detected S-sulfhydration of AAT by H_2_S in both primary HUVECs and primary RPAECs using biotin switch assay. Compared with vehicle group, S-sulfhydration of AAT1 and AAT2 was decreased significantly in both primary endothelial cells of CSE shRNA group, while the supplementation of NaHS significantly reversed the decrease in the S-sulfhydration of AAT (Figures 4C,D). Moreover, NaHS-induced S-sulfhydration of AAT1 and AAT2 in the purified protein from pig heart, which was blocked by the treatment with a thiol reductant DTT (Figure 4E).

Collectively, the above data suggested that H_2_S might inhibit the activity of AAT *via* the sulfhydration of AAT.

### Upregulation of Endogenous SO_2_ Production Exerted Compensatory Effects to Inhibit Inflammation Caused by Downregulated H_2_S/CSE Pathway *In Vitro*

**Figure 5:**
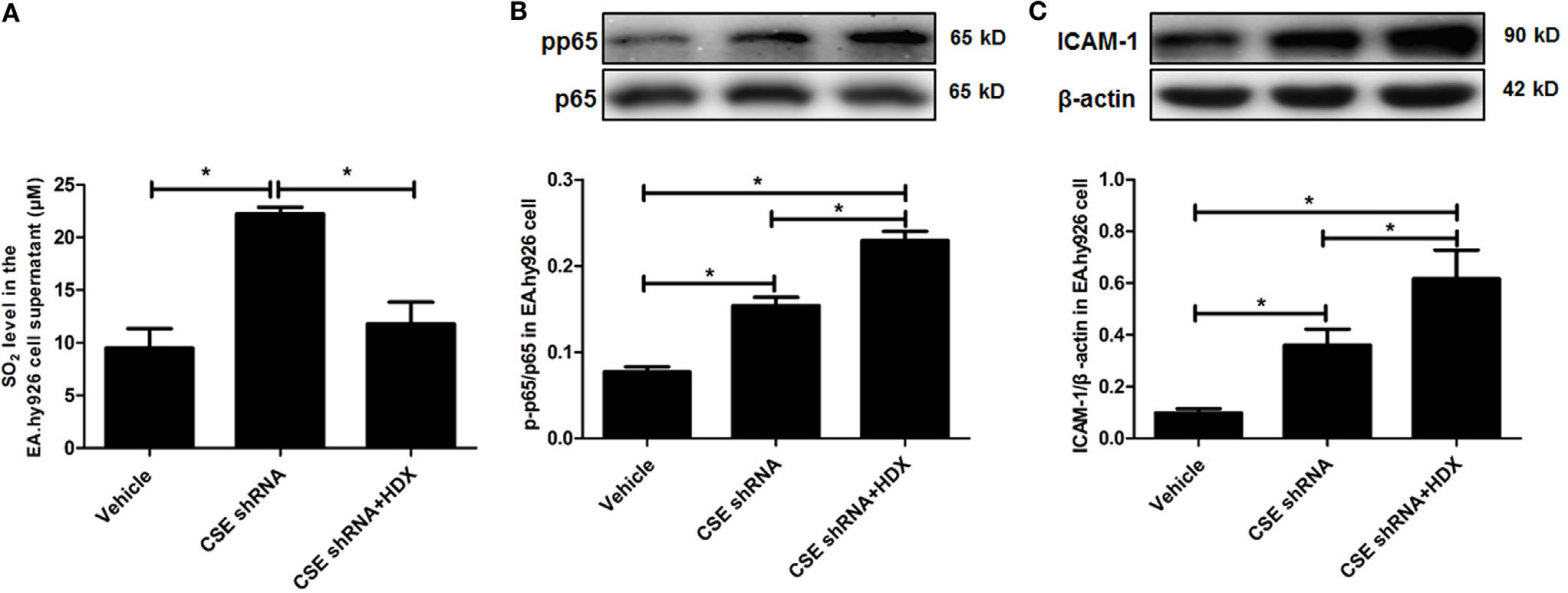
Aspartate aminotransferase inhibitor l-aspartate-β-hydroxamate (HDX) aggravated the inflammation caused by cystathionine-γ-lyase CSE knockdown in EA.hy926 cell. **(A)** The SO_2_ level in the supernatant of EA.hy926 cells detected by HPLC analysis. **(B)** The phosphorylation of NF-κB p65 in the EA.hy926 cell detected by western blot. **(C)** The expression of ICAM-1 in the EA.hy926 cell detected by western blot. *P < 0.05. Data are expressed as means ± SEM, and all experiments were performed independently for at least three times.

In order to explore the biological significance of elevated endogenous SO_2_ levels induced by downregulation of endogenous H_2_S/CSE pathway, CSE knockdown EA.hy926 cells were treated with HDX, an AAT inhibitor. The results showed that compared with vehicle group, the SO_2_ level in the EA.hy926 cell supernatant was sharply increased, and the further treatment by HDX reversed the increased SO_2_ caused by CSE knockdown (Figure 5A). Meanwhile, the phosphorylation of NF-κB p65 (pp65/p65) and the expression of ICAM-1 which denoted the inflammatory response in the EA.hy926 cell were also upregulated by CSE knockdown. However, the treatment of HDX aggravated the increase in the phosphorylation of NF-κB p65 and the expression of ICAM-1, which resulted from the deficiency of endogenous H_2_S/CSE pathway (Figures 5B,C).

**Figure 6:**
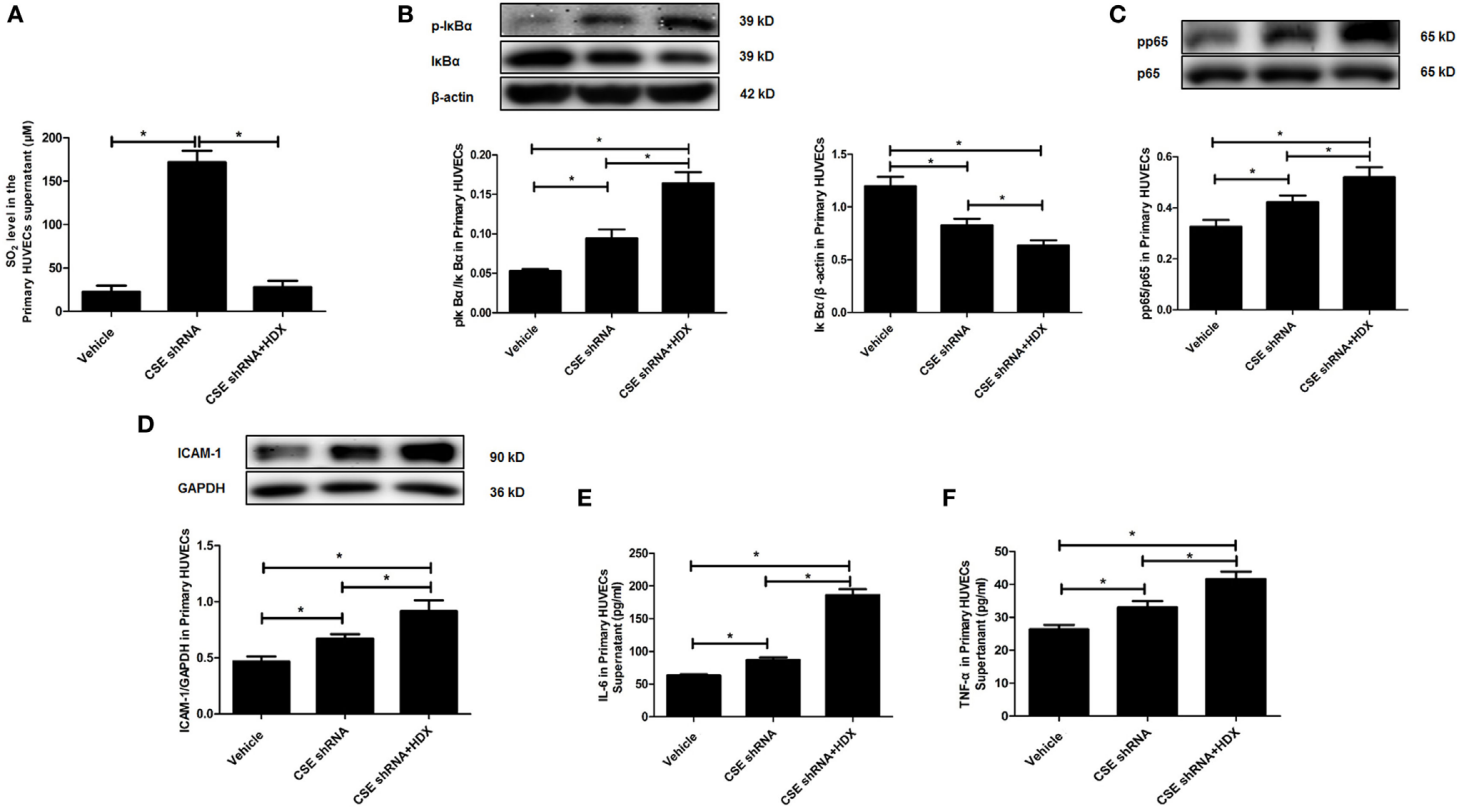
Aspartate aminotransferase inhibitor l-aspartate-β-hydroxamate (HDX) aggravated the inflammation caused by cystathionine-γ-lyase (CSE) knockdown in primary human umbilical vein endothelial cells (HUVECs). **(A)** The SO_2_ level in the supernatant of primary HUVECs detected by high-performance liquid chromatography analysis. **(B)** The phosphorylated and total IκBα in the primary HUVECs detected by western blot. **(C)** The phosphorylation of NF-κB p65 in the primary HUVECs detected by western blot. **(D)** The expression of ICAM-1 in the primary HUVECs detected by western blot. The levels of IL-6 **(E)** and TNF-α **(F)** in the supernatant of the primary HUVECs detected by ELISA. *P < 0.05. Data are expressed as means ± SEM, and all experiments were performed independently for at least three times.

Furthermore, the same protocol of the experiment was done on primary HUVECs. The ratio of phosphorylated IκBα/IκBα (p-IκBα/IκBα), IκBα protein level, the ratio of pp65/p65, and the expression of ICAM-1 were also detected by western blot. The inflammatory cytokines IL-6 and TNF-α in primary HUVEC supernatant were detected by ELISA. Compared with the vehicle group, the SO_2_ level in primary HUVECs was markedly increased by CSE knockdown, and HDX blocked the increase in SO_2_ content in cell supernatant (Figure 6A). The ratio of p-IκBα/IκBα, the ratio of pp65/p65, and the expression of ICAM-1 were all upregulated but IκBα protein level was reduced by CSE knockdown (Figures 6B–D). Meanwhile, the inflammatory cytokines, IL-6 and TNF-α, in primary HUVEC supernatant were elevated by CSE knockdown (Figures 6E,F). However, the treatment of HDX promoted the increase in IκBα and NF-κB p65 phosphorylation and the level of inflammatory cytokines, and aggravated the decrease in IκBα protein level, which resulted from the deficiency of endogenous H_2_S/CSE pathway in the primary HUVECs (Figures 6B–F).

**Figure 7:**
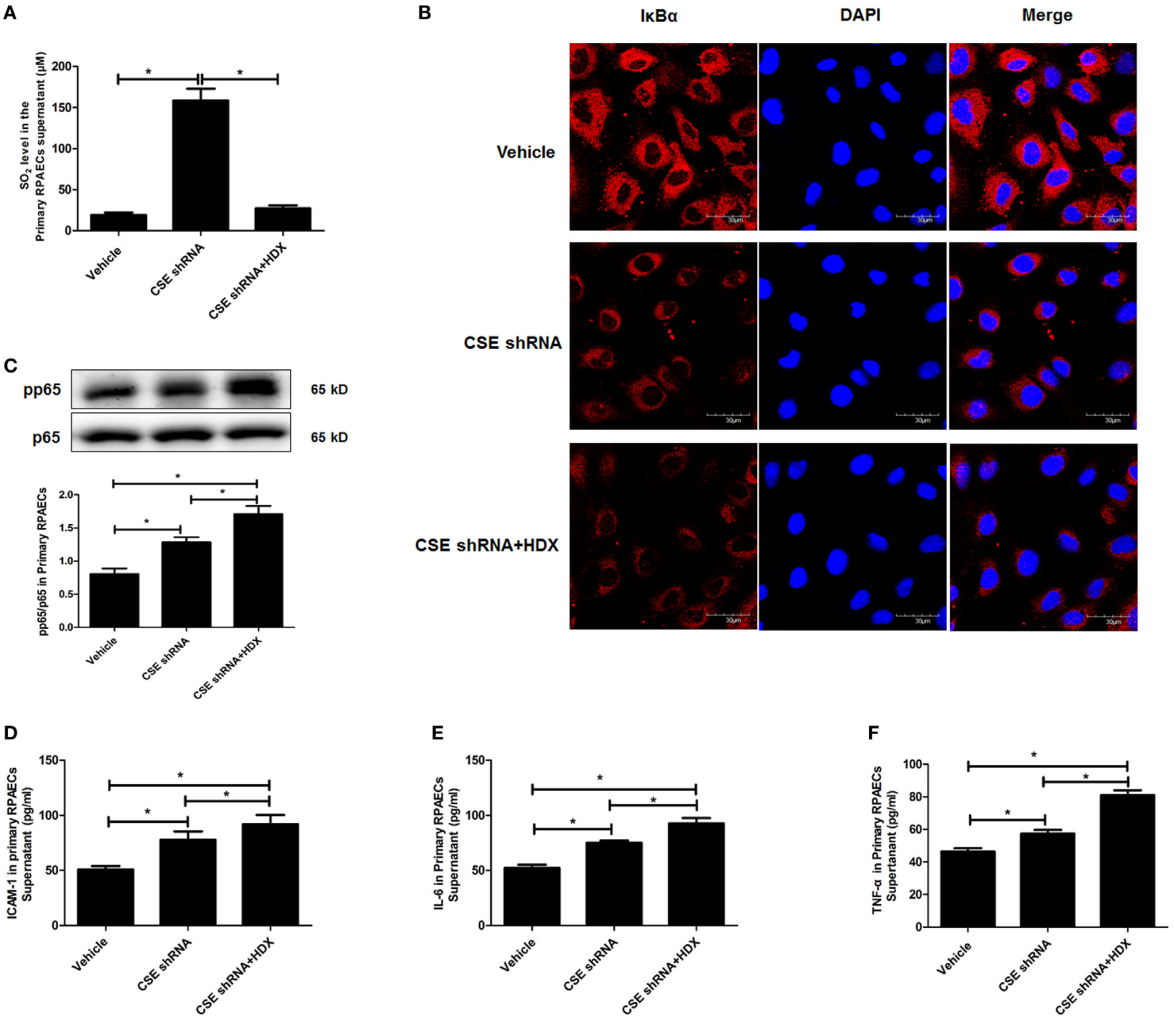
Aspartate aminotransferase inhibitor l-aspartate-β-hydroxamate (HDX) aggravated the inflammation caused by cystathionine-γ-lyase (CSE) knockdown in primary rat pulmonary artery endothelial cells (RPAECs). **(A)** The SO_2_ level in the supernatant of primary RPAECs detected by HPLC analysis. **(B)** The expression of IκBα in the primary RPAECs detected by western blot. **(C)** The phosphorylation of NF-κB p65 in the primary RPAECs detected by western blot. The level of ICAM-1 **(D)**, IL-6 **(E)**, and TNF-α **(F)** in the supernatant of the primary RPAECs detected by enzyme-linked immunosorbent assay. *P < 0.05. Data are expressed as means ± SEM, and all experiments were performed independently for at least three times.

The results observed in the following primary RPAECs were in accordance with those in both EA.hy926 cells and primary HUVECs (Figure 7). HDX inhibited the increased SO_2_ content in the supernatant of primary RPAECs but aggravated the decrease in IκBα protein level and the increase in the phosphorylation of p65 and the levels of ICAM-1, IL-6, and TNF-α in cell supernatants induced by CSE knockdown.

Collectively, the above data implied that the upregulated endogenous SO_2_ production might exert compensatory effects to inhibit the inflammation caused by H_2_S/CSE deficiency.

### Upregulation of Endogenous SO_2_ Production Exerted Compensatory Effects to Inhibit Pulmonary Vascular Inflammation Caused by Downregulated H_2_S/CSE Pathway *In Vivo*

**Figure 8:**
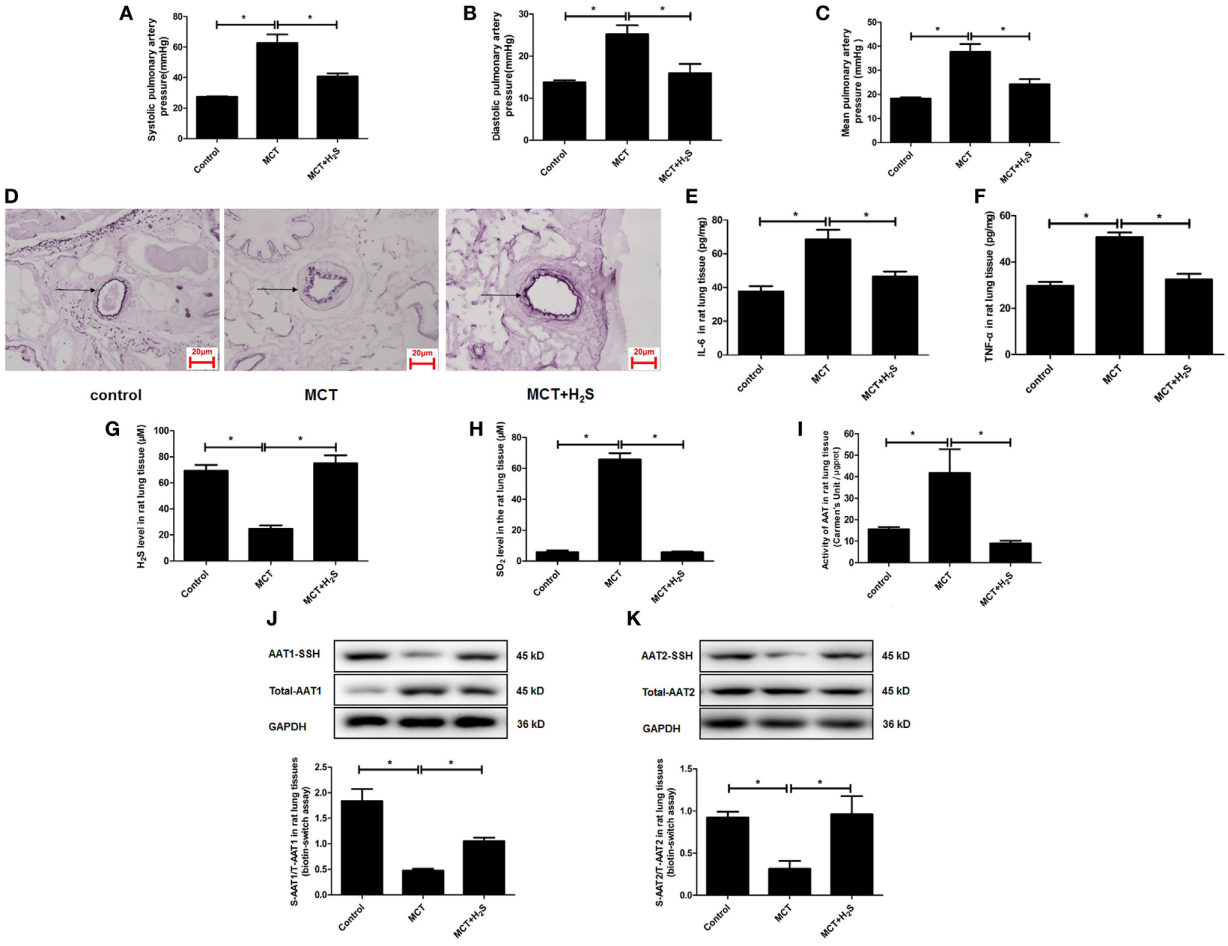
The endogenous sulfur dioxide (SO_2_)/aspartate aminotransferase (AAT) pathway was upregulated by the downregulated H_2_S production in the rats with pulmonary vascular inflammation. **(A–C)** The systolic pulmonary artery pressure, diastolic pulmonary artery pressure and mean pulmonary artery pressure of rats measured by right heart catheterization. **(D)** The morphological change of small pulmonary arteries in the rats observed following the staining by the modified Weigert’s elastic fiber dye. The small pulmonary artery was indicated by dark arrow. The internal and external elastic lamina were shown as dark-purple color under microscope. **(E,F)** The level of IL-6 and TNF-α in the rat lung tissue detected by enzyme-linked immunosorbent assay. **(G)** The H_2_S level in the rat lung tissues detected by a an H_2_S-selective sensor. **(H)** The SO_2_ level in the rat lung tissues detected by HPLC assay. **(I)** AAT activity in the rat lung tissues detected by colorimetry assay. **(J,K)** Sulfhydration of AAT1 and AAT2 in the rat lung tissues detected by biotin switch assay. *P < 0.05. Data are expressed as means ± SEM, *n* = 6.

In order to further elucidate the significance of upregulated endogenous SO_2_ production induced by the deficiency of endogenous H_2_S/CSE pathway in the development of vascular inflammation, we constructed a rat model of pulmonary hypertension in which the endogenous H_2_S production was suppressed by MCT stimulation. The data showed that compared with the control group, the systolic, diastolic, and mean pulmonary arterial pressures in the rats of MCT group were increased, respectively (Figures 8A–C). Moreover, the thickened media of small pulmonary artery and the increased inflammatory cytokines IL-6 and TNF-α in the lung tissue in MCT-treated rat were demonstrated (Figures 8D–F). Simultaneously, the H_2_S content in the lung tissue of rats in the MCT group was lower than that of the control group (Figure 8G). The supplement of H_2_S donor NaHS rescued the pulmonary hypertension, pulmonary vascular remodeling and pulmonary vascular inflammation in the rats of MCT group (Figures 8A–F).

In the above rat model, the effect of H_2_S on the endogenous SO_2_/AAT pathway was examined. The results demonstrated that compared with the control group, SO_2_ level, and AAT activity in the lung tissues of rats in the MCT group were increased significantly (Figures 8H,I). Moreover, the sulfhydration of AAT1 and AAT2 in the lung tissues of MCT rats was decreased compared with the control group (Figures 8J,K). Compared with MCT group, SO_2_ level and AAT activity in the lung tissues of the rats in the MCT + H_2_S group were reduced, while sulfhydrated AAT1 and AAT2 were increased (Figures 8H–K), suggesting that the supplement of H_2_S donor NaHS restored the H_2_S level in the lung tissue of MCT rats, and subsequently blocked the upregulation of endogenous SO_2_/AAT pathway.

**Figure 9:**
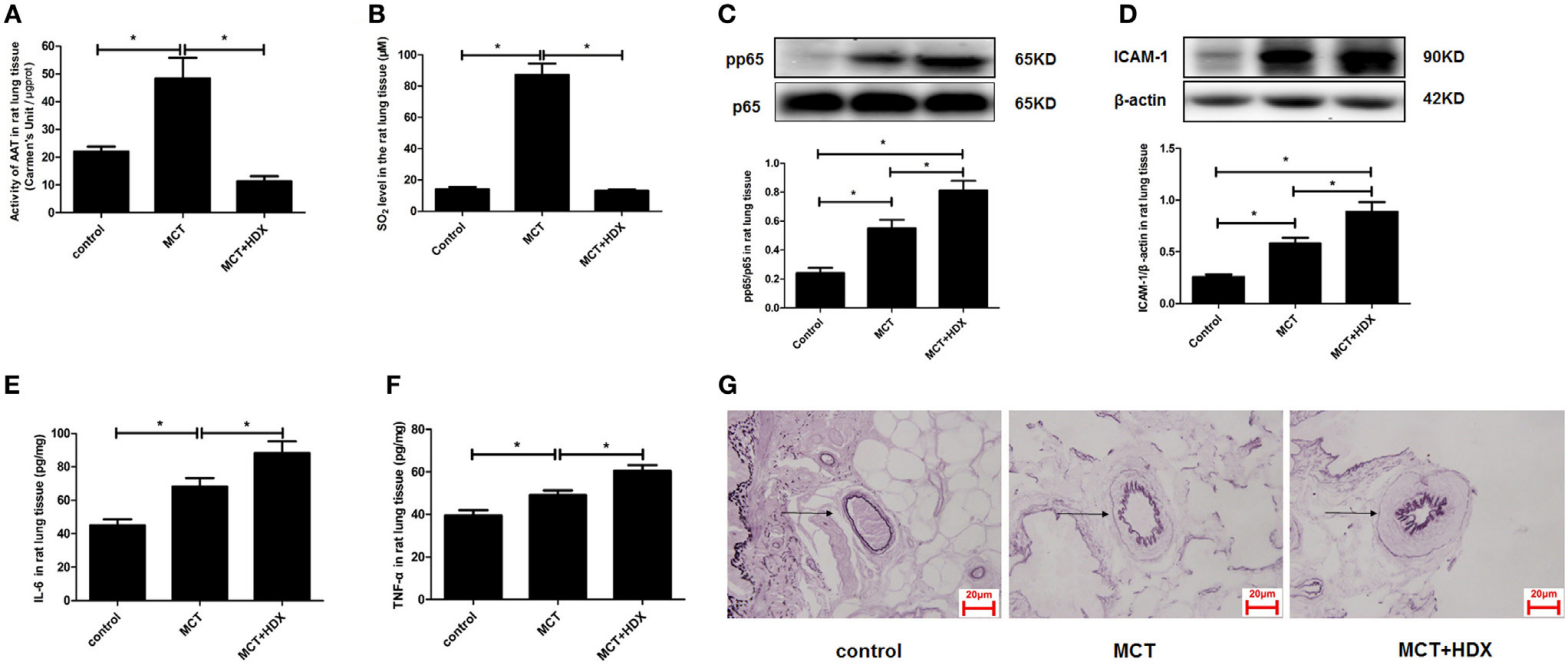
Upregulation of endogenous SO_2_ production exerted compensatory effects to inhibit pulmonary vascular inflammation caused by downregulated H_2_S/cystathionine γ-lyase (CSE) pathway *in vivo*. **(A)** Aspartate aminotransferase (AAT) activity in the rat lung tissues detected by colorimetry assay. **(B)** The SO_2_ level in the rat lung tissues detected by high-performance liquid chromatography assay. **(C)** The ratio of pp65/p65 in the rat lung tissues detected by western blotting. **(D)** The expression of ICAM-1 in the rat lung tissues detected by western blotting. **(E,F)** The level of IL-6 and TNF-α in the rat lung tissue detected by enzyme-linked immunosorbent assay. **(G)** The morphological change of small pulmonary arteries in the rats observed following the staining by the modified Weigert’s elastic fiber dye. The small pulmonary artery was indicated by dark arrow. The internal and external elastic lamina were shown as dark-purple color under microscope. *P < 0.05. Data are expressed as means ± SEM, *n* = 7.

Furthermore, the significance of upregulated SO_2_/AAT pathway in the pulmonary vascular inflammation associated with the downregulation of endogenous H_2_S production was explored in the MCT rats treated with an AAT inhibitor HDX. The data showed that compared with the MCT group, AAT activity and SO_2_ level were suppressed significantly in the lung tissue of rats in the MCT + HDX group (Figures 9A,B). While HDX aggravated the increase in the phosphoralyation of NF-κB p65, ICAM-1 protein expression, the level of IL-6, and TNF-α in the lung tissue of MCT-treated rats (Figures 9C–F). In addition, the thickened media of small pulmonary artery in MCT-treated rats was exacerbated by HDX (Figure 9G), suggesting that the upregulation of endogenous SO_2_ pathway might be an important compensatory response when the endogenous H_2_S pathway collapsed in the development of pulmonary vascular inflammation and pulmonary vascular remodeling.

## Discussion

The impaired H_2_S/CSE pathway was one of important pathogenesis of many cardiovascular diseases due to the lack of protective effect of endogenous H_2_S on the heart and vessel. The facts that CSE knockout mice exhibited a series of marked cardiovascular pathological phenotypes further supported the significance of endogenous H_2_S/CSE in the cardiovascular regulation and diseases. For example, Yuan et al. found that vascular endothelial growth factor (VEGF)-induced vascular solute hyperpermeability was blunted in the CSE gene deficient mice, suggesting that endothelium-derived H_2_S protected the endothelial solute barrier function (44). Mani et al. discovered that CSE gene depletion promoted aortic intimal proliferation and accelerated atherosclerotic development in the ApoE knockout mice fed with atherogenic diet (45). CSE knockout mice were also found to exhibit a delayed wound healing and a markedly reduced microvessel formation in response to VEGF (46). In the present study, we observed the variation of endogenous SO_2_/AAT pathway, another protector in the cardiovascular system, in a CSE knockdown endothelial cell model and further explored its pathological significance in the endothelial cell inflammation.

Firstly, we observed the change of endogenous SO_2_ generation in CSE knockdown EA.hy926 cells using SO_2_ fluorescent probe. The results showed that endogenous SO_2_ level in the CSE knockdown endothelial cells was markedly higher than that in the vehicle endothelial cells, while H_2_S level in the culture supernatant and endothelial cells of CSE shRNA group was decreased compared with vehicle group. Moreover, H_2_S donor NaHS raised the H_2_S level in the supernatant and endothelial cells of CSE shRNA group, and blocked the increase in the SO_2_ level caused by CSE knockdown. In accordance with the results obtained from the HUVEC line, the levels of SO_2_ in the primary HUVECs and RPAECs were also increased by the impaired H_2_S/CSE pathway, while the restoration of H_2_S content in the primary endothelial cells abolished the increase in the endogenous SO_2_ generation. The abovementioned data confirmed that the endogenous H_2_S inhibited endothelium-derived SO_2_ production.

Aspartate aminotransferase is regarded as the key enzyme generating endogenous SO_2_ in the mammal animals. There are two kinds of AAT isoenzymes: AAT1 locates in the cytoplasm and AAT2 in the mitochondria (18, 47). Considering that the expression and activity of AAT are the major elements to control the endogenous SO_2_ production (48), we measured the expression and activity of AAT in the CSE knockdown EA.hy926 cells to explore the mechanism by which endothelium-derived H_2_S repressed endogenous SO_2_ generation. The western blot results showed that there was no difference in the expression of AAT1 and AAT2 in the EA.hy926 cells between vehicle group and CSE shRNA group, suggesting endogenous H_2_S did not affect the expression of AAT protein. We further investigated the role of endogenous H_2_S in the control of AAT activity. Interestingly, the enzymatic activity of AAT in the CSE shRNA endothelial cells was higher than that in the vehicle endothelial cells, while H_2_S donor supplement alleviated the enhancement of AAT activity induced by CSE knockdown. The discrete regulation of the AAT protein expression and activity by H_2_S in the EA.hy926 cells was completely reproduced in both primary endothelial cells. Moreover, in *in vitro* experiment, H_2_S donor was found to directly inhibit activity of purified AAT protein in a concentration-dependent manner, which further supported the speculation that endogenous H_2_S suppressed endothelium-derived SO_2_ generation *via* inhibiting AAT activity. In fact, the detached regulation of the AAT expression and activity was reported, although the expression and activity of AAT were identically controlled in general. For example, Barouki et al. found that the regulation of AAT1 mRNA in the Fao rat hepatoma cell line by dexamethasone correlated with the variation of the AAT1 activity, suggesting that dexamethasone acted at the transcriptional level (49). However, cortisol acetate treatment did not alter AAT1 activity but reduced AAT1 mRNA in rat muscles (50). Therefore, we supposed that the discrete effects of H_2_S on the AAT1/2 protein expression, and activity might result from the fact that H_2_S regulated SO_2_/AAT at a posttranslational level.

Secondly, we tested how H_2_S inhibited AAT activity. It is well known that endogenous H_2_S regulates various cellular processes *via* S-sulfhydration of target proteins, a posttranslational modification at the thiol group in the cysteine residue in the proteins such as Keap1, P66Shc, and NF-κB (1, 33, 42, 51–53), while the thiol group in the cysteine residue is also the molecular target of redox regulation. Coincidentally, AAT expression and activity were controlled in an oxygen-related manner in a rodent model of acute ischemic stroke (54). In the present study, we found that DTT, a thiol reductant, could reverse the H_2_S-induced decrease in the AAT activity in the *in vitro* experiment, suggesting the thiol group might be involved in the regulation of the AAT activity by H_2_S. Therefore, we detected the sulfhydration of AAT1 and AAT2 in the CSE knockdown EA.hy926 cells using the modified biotin switch assay (33). The data showed that CSE knockdown reduced the sulfhydration of AAT1 and AAT2 in the endothelial cells, while H_2_S donor enhanced the sulfhydration of AAT1 and AAT2 in the EA.hy926 cells of CSE shRNA group. To confirm the fact that endogenous H_2_S sulfhydrated AAT protein, we used biotin thiol assay (43), another method for detecting sulfhydration, to investigate the modification of AAT by H_2_S. The change of sulfhydration of AAT detected by biotin thiol assay was similar to the results using modified biotin switch assay. Moreover, in the *in vitro* experiments we discovered that NaHS induced a marked sulfhydration of AAT1 and AAT2, which was blocked by DTT treatment. In the experiment on the primary HUVECs and RPAECs, the decrease in the sulfhydration of AAT1 and AAT2 caused by CSE knockdown was also rescued by NaHS. Those data suggested that sulfhydration of AAT might mediate the inhibitory effect of endogenous H_2_S on the AAT activity.

On the basis of H_2_S/CSE deficiency-induced inflammation endothelial cell model, we further investigated the pathological significance of CSE knockdown-enhanced endogenous SO_2_ production. We used HDX, an AAT inhibitor, to block the increased endogenous SO_2_ production in the HUVEC line, primary HUVECs and RPAECs, and observed the changes of NF-κB pathway, a pivot regulator of cellular inflammation, and its downstream target genes including inflammatory cytokine ICAM-1, IL-6, and TNF-α. NF-κB, consisting of p65 and p50 subunits, locates in the cytosol complexed with the inhibitory protein IκBα in an inactivated state. Inflammatory stimuli such as hypoxia can activate the phosphorylation of IκBα, leading to the IκBα degradation and dissociation from NF-κB. The released NF-κB is subsequently phosphorylated, translocates into the nucleus and increases the transcription of inflammatory cytokines (33). ICAM-1 is typically expressed on the surface of endothelial cells and of other inflammatory cells and mediates the binding of leukocytes to endothelial cell by coupling its ligand integrin. ICAM-1 is regarded as a classical marker of endothelial inflammation (55). Therefore, we detected the phosphorylated IκBα, total IκBα, phosphorylated NF-κB p65, and ICAM-1 protein in the endothelial cells and ICAM-1, IL-6, and TNF-α levels in the supernatant to reflect the endothelial cell inflammation. As we expected, HDX aggravated the increase in the expression of ICAM-1 and the phosphorylation of NF-κB p65 in the CSE knockdown EA.hy926 cells. Moreover, HDX was found to promote phosphorylation of IκBα, decrease IκBα protein level, and raise the phosphorylated NF-κB p65 in both primary endothelial cells. The effects of HDX on the inflammatory cytokines in the supernatant of primary endothelial cells were in line with the regulation on the NF-κB pathway. Therefore, we supposed that endogenous SO_2_/AAT pathway was upregulated as a compensatory mechanism for the downregulated endogenous H_2_S pathway in the endothelial cell inflammation.

Finally, we further explored the importance of upregulated SO_2_/AAT pathway following the broken H_2_S/CSE pathway in the *in vivo* experiments. As previously reported, endogenous H_2_S production in the rat lung tissues was downregulated by MCT treatment in a rat model of pulmonary vascular inflammation (5). Conversely, SO_2_ content and AAT activity in the lung tissue of MCT-treated rats were enhanced, while the AAT1 and AAT2 sulfhydraton was reduced. More interestingly, the restoration of H_2_S level reversed the upregulation of endogenous SO_2_/AAT pathway, demonstrated by the facts that NaHS increased the sulfhydrated AAT1 and AAT2, inactivated the AAT activity and reduced SO_2_ level in the lung tissue of rats in the MCT groups. Furthermore, as designed in the endothelial cell experiments, we used HDX and found that it inhibited the upregulation of endogenous SO_2_/AAT pathway. As we expected, the pulmonary vascular inflammation reflected by the phosphorylation of NF-κB p65 and the elevated inflammatory cytokines including ICAM-1, IL-6, and TNF-α was exacerbated when the deficient H_2_S-induced SO_2_/AAT pathway was blocked by HDX. Moreover, in our previous studies, HDX was also found to exacerbate the MCT-induced pulmonary vascular inflammation, demonstrated by the fact that HDX enhanced NF-κB p65 and ICAM-1 expression in the pulmonary artery endothelial cells in an immunohistochemical study (56). As a result, HDX aggravated the thickened media of pulmonary artery in the MCT-treated rats in accordance with the findings previously reported (56, 57). The abovementioned results were in accordance with the data obtained from *in vitro* endothelial cell experiment. Therefore, we supposed that endogenous SO_2_/AAT pathway was upregulated as a compensatory mechanism for the downregulated endogenous H_2_S pathway in the endothelial cell inflammation.

In brief, we firstly demonstrated that endogenous H_2_S inhibited endothelial cell-derived SO_2_ generation through suppressing AAT activity *via* sulfhydration *in vitro* and *in vivo*. When injury factors impaired H_2_S/CSE pathway, the endogenous SO_2_ production was subsequently induced as a reserved protector to protect the endothelial cell functions such as anti-inflammatory effects. Our findings deepen the understanding of regulatory mechanism responsible for cardiovascular homeostasis, providing a new insight for the exploration of interaction among bioactive small molecules. More molecular and cellular biological studies, however, need to be done for disclosing the precise target and mechanisms by which endogenous H_2_S functions.

## Ethics Statement

This study was carried out in accordance with the Animal Management Rule of the Ministry of Health of the People’s Republic of China. The protocol was approved by the Animal Research Ethics Committee of Peking University First Hospital.

## Author Contributions

DZ, XW, XT and CL carried out the experimental work. DZ wrote the paper. HJ and YH designed and supervised the experiments. HJ, KL, XY and XT revised the primary manuscript. JZ, WK, JD and CT were responsible for the quality control and analysis. DZ, LZ, GY and YT participated in the data analysis. All authors approved the final version of the manuscript.

## Conflict of Interest Statement

The authors declare that the research was conducted in the absence of any commercial or financial relationships that could be construed as a potential conflict of interest.

## Supplementary Material

Figure S1 Transfection of cystathionine-γ-lyase (CSE) shRNA lentivirus downregulated the H_2_S/CSE pathway in EA.hy926 cell. The EA.hy926 human umbilical vein endothelial cells (HUVECs) were, respectively, transfected with lentivirus containing CSE shRNA plus green fluorescent protein (GFP) cDNA at different concentrations, 1 × 10^4^, 2 × 10^4^, 4 × 10^4^, 1 × 10^5^, and 2 × 10^5^ TU/mL to determine the appropriate concentration. **(A)** The GFP green fluorescence observed in the infected EA.hy926 cells after 72 h under fluorescence microscope. The images showed that more than 90% of cells showed green fluorescence at the lentivirus concentration of 1 × 10^5^ and 2 × 10^5^ TU/mL compared with control group, suggesting that the lentivirus was successfully transfected. **(B)** The expression of CSE in the EA.hy926 cells detected by western blot. Compared with the control group, the expression of CSE was decreased by 60.2% and 65.1% at the lentivirus concentration of 1 × 10^5^ and 2 × 10^5^ TU/mL. **(C)** The H_2_S level in the supernatant of EA.hy926 cells was detected by H_2_S-selective sensor. Compared with control group, the H_2_S level in EA.hy926 cell supernatant was significantly decreased by 63.6% and 75%, respectively, which was similar to the change of CSE expression. * *P* < 0.05. Data are expressed as means ± SEM, and all experiments were performed independently for at least three times.

Figure S2 The schematic protocol of biotin thiol assay to detect the S-sulfhydration. The sample protein was incubated with maleimide-PEG2-biotin to alkylate both cysteine residue and sulfhydrated cysteine residue at the first step. At the subsequent step, the high-capacity affinity streptavidin–agarose resin was used to pull down the proteins which were alkylated by maleimide-PEG2-biotin at the first step. At the last step, the proteins were reacted with buffer with or without DTT, a thiol reductant, for 30 min followed by centrifugation. DTT was used for cleaving the mixed disulfide bond and releasing the sulfhydrated protein. Therefore, sulfhydrated protein separated from the mixture containing DTT was detected by western blot, while supernatant separated from the mixture without DTT was used as negative control.
