# Supplementary figures and images for: The Increased Endogenous Sulfur Dioxide Acts as a Compensatory Mechanism for the Downregulated Endogenous Hydrogen Sulfide Pathway in the Endothelial Cell Inflammation
Source: Front Immunol. 2018 Apr 30;9:882. doi: 10.3389/fimmu.2018.00882 (PMC5936987; doi:10.3389/fimmu.2018.00882)

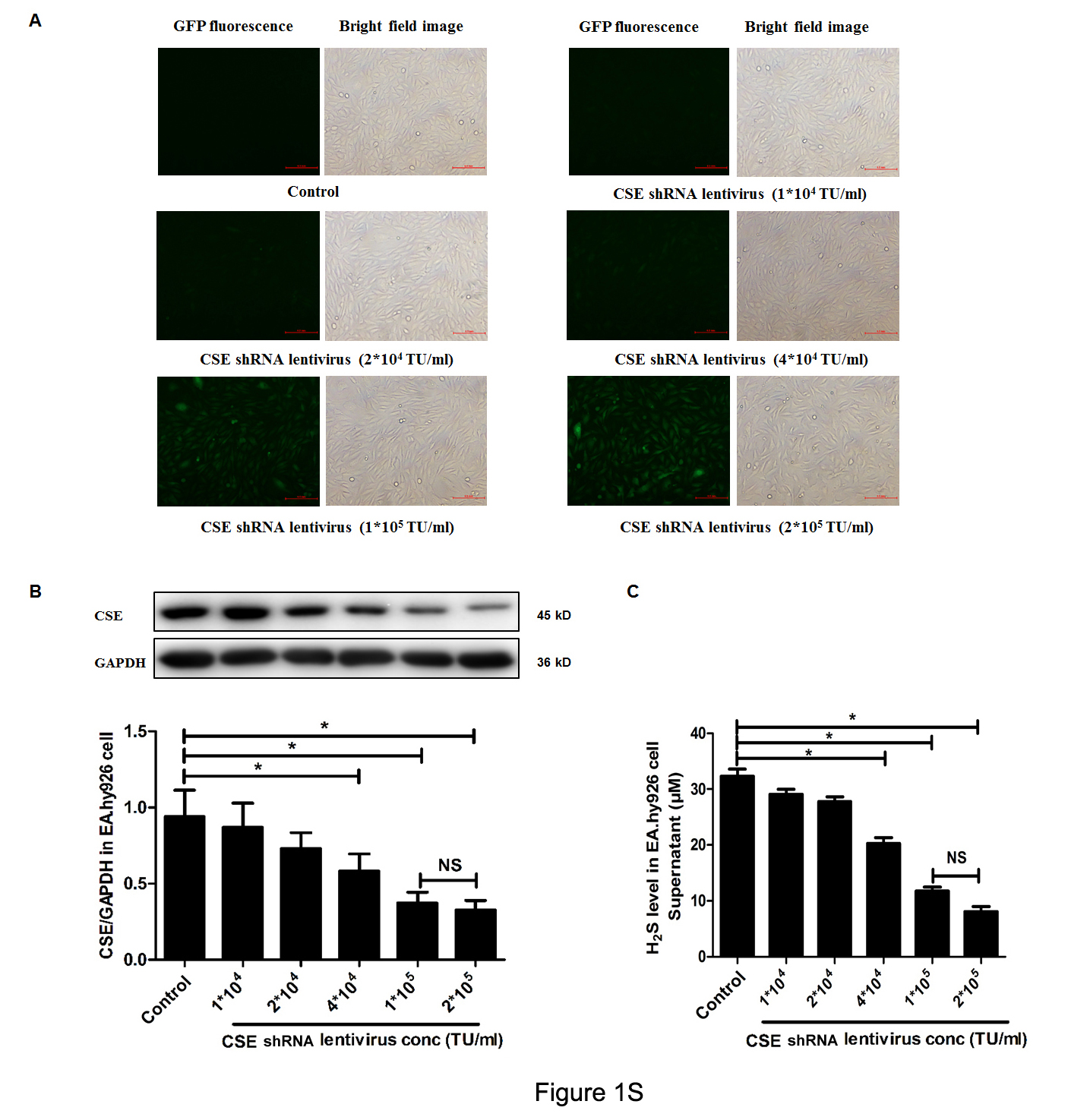

Supplement: Figure S1 — Transfection of cystathionine-γ-lyase (CSE) shRNA lentivirus downregulated the H2S/CSE pathway in EA.hy926 cell. The EA.hy926 human umbilical vein endothelial cells (HUVECs) were, respectively, transfected with lentivirus containing CSE shRNA plus green fluorescent protein (GFP) cDNA at different concentrations, 1 × 10^4^, 2 × 10^4^, 4 × 10^4^, 1 × 10^5^, and 2 × 10^5^ TU/mL to determine the appropriate concentration. (A) The GFP green fluorescence observed in the infected EA.hy926 cells after 72 h under fluorescence microscope. The images showed that more than 90% of cells showed green fluorescence at the lentivirus concentration of 1 × 10^5^ and 2 × 10^5^ TU/mL compared with control group, suggesting that the lentivirus was successfully transfected. (B) The expression of CSE in the EA.hy926 cells detected by western blot. Compared with the control group, the expression of CSE was decreased by 60.2% and 65.1% at the lentivirus concentration of 1 × 10^5^ and 2 × 10^5^ TU/mL. (C) The H2S level in the supernatant of EA.hy926 cells was detected by H2S-selective sensor. Compared with control group, the H2S level in EA.hy926 cell supernatant was significantly decreased by 63.6% and 75%, respectively, which was similar to the change of CSE expression. * P < 0.05. Data are expressed as means ± SEM, and all experiments were performed independently for at least three times. [file image_1.jpeg]

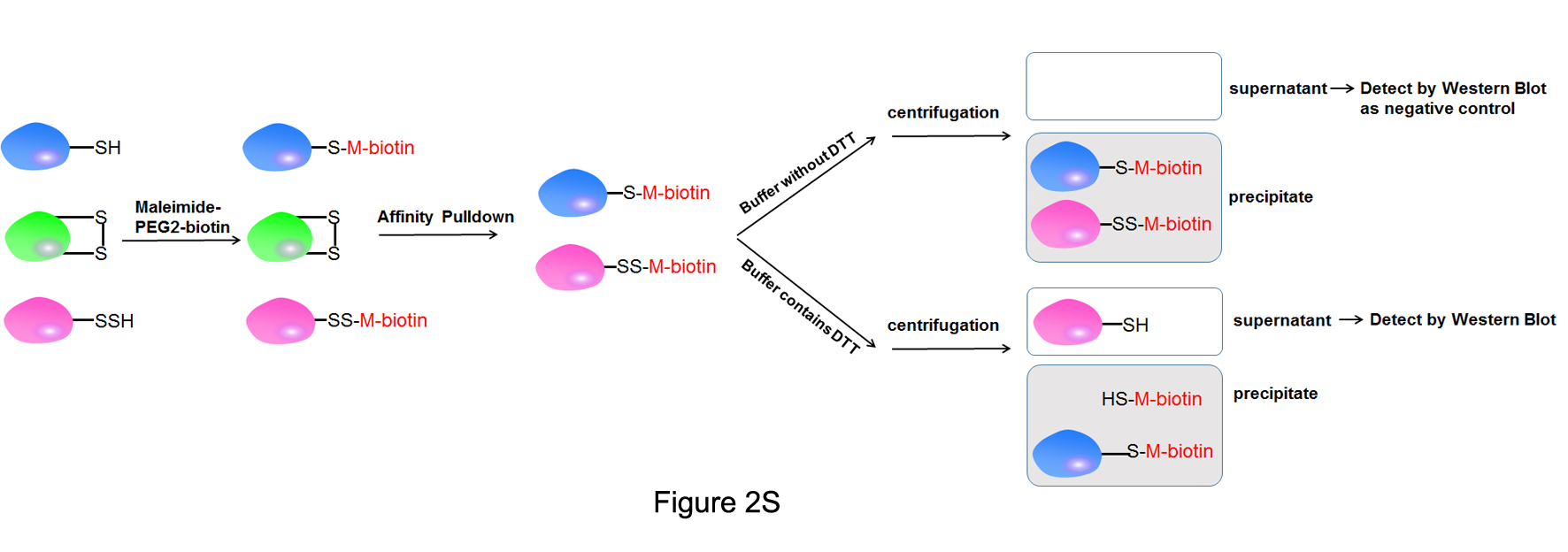

Supplement: Figure S2 — The schematic protocol of biotin thiol assay to detect the S-sulfhydration. The sample protein was incubated with maleimide-PEG2-biotin to alkylate both cysteine residue and sulfhydrated cysteine residue at the first step. At the subsequent step, the high-capacity affinity streptavidin–agarose resin was used to pull down the proteins which were alkylated by maleimide-PEG2-biotin at the first step. At the last step, the proteins were reacted with buffer with or without DTT, a thiol reductant, for 30 min followed by centrifugation. DTT was used for cleaving the mixed disulfide bond and releasing the sulfhydrated protein. Therefore, sulfhydrated protein separated from the mixture containing DTT was detected by western blot, while supernatant separated from the mixture without DTT was used as negative control. [file image_2.jpeg]
